# Supplementary material for: More Adult Women than Men at High Cardiometabolic Risk Reported Worse Lifestyles and Self-Reported Health Status in the COVID-19 Lockdown
Source: Nutrients. 2024 Jun 24;16(13):2000. doi: 10.3390/nu16132000 (PMC11243243; doi:10.3390/nu16132000)
Supplement: Supplementary file 1 [file nutrients-16-02000-s001.zip › nutrients-3072845-supplementary.pdf]

Table S1. Questions analysed in the present study

Variable	Question	Answer options	Categories in analyses
MedDiet	Do you think your adherence to the healthy Mediterranean diet that we are recommending in this study has changed during your confinement?	It has improved a lot<br>It has improved a little<br>It has not changed<br>It has got a little worse<br>It has got much worse	Improved a lot/a little<br>Not changed<br>A little/much worse
PA	Has your physical activity level changed during confinement?	More physical activity<br>Less physical activity<br>No	More physical activity<br>Less physical activity<br>No
Sleep	On average, how many hours did you sleep at night during confinement?	Less than 6 hours<br>6 - 7 hours<br>8 - 9 hours<br>More than 9 hours	Less than 6 hours<br>6 - 7 hours<br>8 - 9 hours<br>More than 9 hours
	How would you rate the quality of your sleep during confinement?	Very good<br>Fairly good<br>Average<br>Fairly bad<br>Very bad	Very/Fairly good<br>Average<br>Fairly/very bad
	How would you rate your sleep quality during confinement compared to your usual sleep quality?	Better than usual<br>Worse than usual<br>Same as usual	Better than usual<br>Worse than usual<br>Same as usual
SRH	How would you rate your health during confinement?	Excellent<br>Very good<br>Good<br>Fair<br>Poor	Excellent/Very good<br>Good<br>Fair/Poor
	How would you say your health is now compared to before confinement?	Much better<br>A little better<br>About the same<br>A little worse<br>Much worse	Much/a little better<br>About the same<br>A little/much worse

Abbreviations: PA, physical activity; SRH, self-reported health.
